# Role of Kekulé and Non-Kekulé Structures in the Radical Character of Alternant Polycyclic Aromatic Hydrocarbons: A TAO-DFT Study

**DOI:** 10.1038/srep30562

**Published:** 2016-07-26

**Authors:** Chia-Nan Yeh, Jeng-Da Chai

**Affiliations:** 1 Department of Physics, National Taiwan University, Taipei 10617, Taiwan; 2 Center for Theoretical Sciences and Center for Quantum Science and Engineering, National Taiwan University, Taipei 10617, Taiwan

## Abstract

We investigate the role of Kekulé and non-Kekulé structures in the radical character of alternant polycyclic aromatic hydrocarbons (PAHs) using thermally-assisted-occupation density functional theory (TAO-DFT), an efficient electronic structure method for the study of large ground-state systems with strong static correlation effects. Our results reveal that the studies of Kekulé and non-Kekulé structures qualitatively describe the radical character of alternant PAHs, which could be useful when electronic structure calculations are infeasible due to the expensive computational cost. In addition, our results support previous findings on the increase in radical character with increasing system size. For alternant PAHs with the same number of aromatic rings, the geometrical arrangements of aromatic rings are responsible for their radical character.

Polycyclic aromatic hydrocarbons (PAHs), which consist of three or more aromatic rings, are molecules made of carbon and hydrogen atoms only. While some PAHs possess non-radical (i.e., closed-shell) character, many others can exhibit radical (i.e., open-shell or multi-reference) character due to the delocalized nature of *π*-electrons. PAHs with multi-reference ground states, which belong to strongly correlated systems, have been actively studied, owing to their attractive properties and applications^1^,^2^,^3^,^4^,^5^,^6^,^7^,^8^,^9^,^10^,^11^.

In particular, alternant PAHs, which are PAHs constituted only of six-membered rings, are planar molecules. Consequently, alternant PAHs have been frequently taken as finite-size models of hydrogen-passivated graphene nanoribbons and nanoflakes. Experimentally, in spite of the promising properties of alternant PAHs, their instability is a commonly encountered problem. Because of their high reactivity, alternant PAHs with substantial radical character are usually short-lived. Methods for stabilization are necessary for their practical use. Theoretically, due to the multi-reference character of ground-state wavefunctions, alternant PAHs with significant radical character cannot be adequately described by conventional electronic structure methods^12^,^13^,^14^,^15^,^16^,^17^,^18^,^19^,^20^,^21^,^22^,^23^,^24^,^25^,^26^,^27^,^28^,^29^,^30^, including the very popular Kohn-Sham density functional theory (KS-DFT)^31^ with commonly used semilocal^32^, hybrid^33^,^34^,^35^,^36^,^37^,^38^, and double-hybrid^39^,^40^,^41^,^42^ density functionals.

High-level *ab initio* multi-reference methods^18^,^21^,^24^,^26^,^28^,^43^,^44^ are typically required to accurately describe the electronic properties of alternant PAHs with pronounced radical character, and their radical character may be examined from the analysis of the natural orbital occupation numbers (NOONs) [i.e., the eigenvalues of one-electron reduced density matrix]^18^,^21^,^24^,^26^,^28^,^45^,^46^. For a molecule with a singlet ground state, if some of the NOONs deviate strongly from the closed-shell values of two/zero, the molecule exhibits pronounced radical character (e.g., diradical, tetraradical, or higher-order radical character). On the other hand, if all the NOONs are close to the closed-shell values of two/zero, the molecule has non-radical character.

Figure 1 shows three alternant PAHs: hexacene, superbenzene, and triangulene, all of which consist of six aromatic rings. To assess their radical character, Pelzer *et al*.^21^ calculated the occupation numbers of the highest occupied (*n*_HONO_) and lowest unoccupied (*n*_LUNO_) natural orbitals for the lowest singlet states of these molecules, using the active-space variational two-electron reduced-density-matrix (RDM-CASSCF) method^43^. Their results revealed that triangulene (*n*_HONO_ = 1.095 and *n*_LUNO_ = 0.916) exhibits strong radical character, hexacene possesses partial radical character (*n*_HONO_ = 1.542 and *n*_LUNO_ = 0.472), and superbenzene (*n*_HONO_ = 1.818 and *n*_LUNO_ = 0.204) has a stable singlet ground state. As these molecules all contain six aromatic rings, it seems plausible that the geometrical arrangements of aromatic rings may be responsible for the radical character of alternant PAHs. Arguments in support of this are also available in several studies^15^,^18^,^20^,^22^,^24^,^25^,^26^,^28^,^29^,^43^. However, owing to the prohibitively high cost of accurate multi-reference calculations, there have been very few studies on large alternant PAHs (e.g., containing up to a few thousand electrons).

In order to extend the investigation to include large alternant PAHs, an efficient electronic structure method for the study of large ground-state systems with strong static correlation effects should be adopted. Recently, we have developed thermally-assisted-occupation density functional theory (TAO-DFT)^23^,^27^, which can be an ideal electronic structure method for the study of large alternant PAHs^29^. Besides, the orbital occupation numbers from TAO-DFT, which are intended to simulate the NOONs, could provide information useful in assessing the possible radical character of alternant PAHs.

In this work, we first show that the orbital occupation numbers from TAO-DFT are qualitatively similar to the NOONs from RDM-CASSCF on a set of small alternant PAHs. Next, the orbital occupation numbers from TAO-DFT are adopted to examine the radical character of large alternant PAHs. In addition, we also propose a simple model based on the extended Clar’s rule to qualitatively describe the radical character of alternant PAHs (i.e., without performing electronic structure calculations), which is especially desirable for very large alternant PAHs. From this simple model, we can easily illustrate how the geometrical arrangements of aromatic rings are responsible for the radical character of the alternant PAHs with the same number of aromatic rings.

## Simple Model Based on the Extended Clar’s Rule

The bonding patterns in benzene can be understood by the resonance between the two Kekulé structures of benzene^47^. The superposition of these two structures, Clar’s aromatic sextet^48^, can be interpreted as six *π*-electrons moving all around the aromatic ring. For alternant PAHs, more than two Kekulé structures may, however, be needed to describe the resonance. Clar’s rule states that the Kekulé structure with the largest number of disjoint aromatic sextets (i.e., benzene-like moieties), the Clar structure, is the most important structure for the characterization of the properties of PAHs^48^,^49^,^50^,^51^. However, the studies of Kekulé structures alone cannot adequately describe open-shell singlet states. Therefore, recent studies have extended Clar’s rule to include the non-Kekulé structures (e.g., the structures with unpaired *π*-electrons)^52^,^53^,^54^. Accordingly, to describe the resonance in alternant PAHs, all the Kekulé and non-Kekulé structures can be adopted (see Fig. 2). For alternant PAHs with strong radical character, the non-Kekulé structures are expected to be essential.

Nevertheless, in the extended Clar’s rule, the relationship between the non-Kekulé structures and radical character of alternant PAHs remains unclear. In previous studies, it has been suggested that the analysis of the radical character of alternant PAHs could be focused solely on the energy balance between the break of a covalent bond and aromatic stabilization^52^,^53^,^54^. In other words, only the energies of the Kekulé and non-Kekulé structures have been previously considered. In this work, we highlight the importance of the degeneracies of the Kekulé and non-Kekulé structures in the radical character of alternant PAHs.

Here we propose a simple model to qualitatively describe the radical character of alternant PAHs. To start with, we define the standard structure of molecule *α* as the structure:

1. Which has no aromatic sextet
2. Whose *π*-electrons pair with their nearest neighbors to form carbon-carbon (C-C) double bonds, yielding the least number of unpaired *π*-electrons.

Figure 3 illustrates the standard structures of (a) hexacene, (b) superbenzene, and (c) triangulene. Unlike hexacene and superbenzene, there are two unpaired *π*-electrons in the standard structure of triangulene, owing to the peculiar geometry of triangulene.

Next, we assign the energies of the structures of molecule *α*. The energy of the standard structure is assigned as *E*_*α*_ (kJ/mol). As it has been reported that the aromatic stabilization energy of benzene is about 90 kJ/mol^55^, and the C-C *π*-bonding energy is about 272 kJ/mol^56^, the energy of the structure with one extra aromatic sextet is assigned as *E*_*α*_ − 90 (kJ/mol), the energy of the structure with two extra aromatic sextets is assigned as *E*_*α*_ − 90 × 2 (kJ/mol), and so on, while the energy of the structure with two extra unpaired *π*-electrons is assigned as *E*_*α*_ + 272 (kJ/mol), the energy of the structure with four extra unpaired *π*-electrons is assigned as *E*_*α*_ + 272 × 2 (kJ/mol), and so on. By counting the number of extra aromatic sextets and the number of extra unpaired *π*-electrons in a structure, the energy of the structure can be assigned. For brevity, the structures with the lowest, second lowest, and third lowest energies are referred as the most important, second most important, and third most important structures, respectively.

In addition, we count the degeneracies of the structures of molecule *α*. Note that the structures with the same energy (e.g., those with the same number of extra aromatic sextets and the same number of extra unpaired *π*-electrons) are degenerate. Here we demonstrate how to count the degeneracies of the top three most important structures of pentacene (see Fig. 4) for instance. Figure 4(a) shows all the *π*-electrons unpaired (marked with black dots), which may later pair with their nearest neighbors to form C-C double bonds or aromatic sextets, or remain unpaired. Figure 4(b) shows various ways to draw the most important structures (i.e., the structures with one extra aromatic sextet). Clearly, the aromatic sextet can be placed in one of the five aromatic rings. Noteworthy, once the aromatic sextet is placed, there is only one way for the remaining *π*-electrons pairing with their nearest neighbors (i.e., forming C-C double bonds). Therefore, the degeneracy of the most important structure is 5. Figure 4(c) shows the second most important structures (i.e., the structures with two extra aromatic sextets and two extra unpaired *π*-electrons). Since the two aromatic sextets should be placed in two of the five aromatic rings, and they cannot be adjacent, there are five ways to arrange them. Besides, the two unpaired *π*-electrons should be placed accordingly. For example, if the two aromatic sextets are placed in rings 1 and 4, the two unpaired *π*-electrons can only be placed at ring 2 or 3, and they cannot both be placed at the top or bottom. As a result, there are only four ways to arrange these two unpaired *π*-electrons. Therefore, the degeneracy of the second most important structure is 20. Figure 4(d) shows the only way to draw the third most important structure (i.e., the structure with three extra aromatic sextets and four extra unpaired *π*-electrons). As the three aromatic sextets cannot be adjacently placed, they can only be placed in rings 1, 3, and 5. Therefore, the degeneracy of the third most important structure is 1.

Finally, similar to expanding a wavefunction by a linear combination of configuration state functions in the full configuration interaction method, in our simple model, the radical character of molecule *α* is argued to be related to all the possible Kekulé and non-Kekulé structures as well as their corresponding weights. The weight of a structure should be dependent on the energy and degeneracy of the structure. The structure with lower energy and greater degeneracy should have a larger weight, and hence govern the radical character of molecule *α* more significantly, while the structure with higher energy and less degeneracy should have a smaller weight, and hence govern the radical character of molecule *α* less significantly.

## Computational Details

As alternant PAHs are all planar molecules, we define a one-dimensional (1D) alternant PAH as the alternant PAH with aromatic rings connected to no more than two other aromatic rings. An alternant PAH that is not a 1D alternant PAH is defined as a two-dimensional (2D) alternant PAH.

To obtain the ground states of the alternant PAHs studied, TAO-LDA (i.e., TAO-DFT with the local density approximation)^23^ is employed for the lowest singlet and triplet energies of each alternant PAH on the respective geometries that were fully optimized at the same level of theory. The singlet-triplet energy gap (ST gap) of the alternant PAH is calculated as (*E*_T_ − *E*_S_), the energy difference between the lowest triplet (T) and singlet (S) states. For the lowest singlet state of the alternant PAH, the active orbital occupation numbers obtained from TAO-LDA are adopted to assess the radical character of the alternant PAH. Here, the highest occupied molecular orbital (HOMO) is the (*N*/2)^th^ orbital, and the lowest unoccupied molecular orbital (LUMO) is the (*N*/2 + 1)^th^ orbital, with *N* being the number of electrons in the alternant PAH. For all the TAO-LDA calculations, the optimal value of *θ* = 7 mhartree (as defined in ref. 23) is adopted. As the spin-restricted and spin-unrestricted TAO-LDA energies for the lowest singlet states of all the alternant PAHs adopted are essentially the same, and spin-unrestricted TAO-LDA calculations are always performed for the lowest triplet states, we remove the phrases “spin-restricted” and “spin-unrestricted” for brevity.

All calculations are performed with a development version of Q-Chem 4.0^57^. Results are computed using the 6-31G(d) basis set with the fine grid EML(75,302), consisting of 75 Euler-Maclaurin radial grid points and 302 Lebedev angular grid points.

## Results and Discussion

### Validity of TAO-LDA Occupation Numbers

Here we examine the validity of TAO-LDA occupation numbers with respect to the NOONs obtained from the accurate RDM-CASSCF method^21^. As illustrated in Fig. 5, we adopt a test set of 24 alternant PAHs, which are the PAHs (excluding the non-alternant PAHs) studied by Pelzer *et al*.^21^. Based on the TAO-LDA calculations (see Table 1), the ST gaps of the 24 alternant PAHs are positive, so they all possess singlet ground states. Besides, the occupation numbers of the highest occupied (*f*_HOMO_) and lowest unoccupied (*f*_LUMO_) molecular orbitals for the lowest singlet states (i.e., the ground states) of the 24 alternant PAHs, calculated using TAO-LDA are compared with the corresponding NOONs (*n*_HONO_ and *n*_LUNO_, respectively) obtained from RDM-CASSCF^21^. Owing to the limitation of TAO-LDA (with a system-independent *θ* = 7 mhartree), the TAO-LDA occupation numbers are slightly biased towards closed-shell systems. A system-dependent *θ* (related to the distribution of NOONs) is expected to improve the general performance of TAO-LDA^23^. Nevertheless, as shown in Fig. 6, even with a fixed value of *θ* (i.e., 7 mhartree), the TAO-LDA occupation numbers are qualitatively similar to the RDM-CASSCF NOONs, yielding a similar trend for the radical character of the 24 alternant PAHs. Our TAO-LDA results support previous findings on the increase in radical character with increasing system size. For the alternant PAHs with the same number of aromatic rings, the geometrical arrangements of aromatic rings are shown to be responsible for the radical character, and it is observed that the smaller the ST gap, the stronger the radical character. Owing to its computational efficiency and reasonable accuracy, in this work, we adopt the TAO-LDA occupation numbers as the approximate NOONs to assess the radical character of the alternant PAHs studied.

### 1D Alternant PAHs

Figure 7 represents three possible ways to arrange the aromatic rings in 1D alternant PAHs. Here the alternant PAH with armchair edges is denoted as *n*-PP [a planar poly(p-phenylene) oligomer]^30^, the alternant PAH with zigzag edges is denoted as *n*-acene, and the alternant PAH with the sawtooth arrangements of aromatic rings is denoted as *n*-phenacene, where *n* is the number of aromatic rings in the alternant PAH. Based on the TAO-LDA calculations, *n*-PP, *n*-acene, and *n*-phenacene (*n* = 3–20) all possess singlet ground states (see Table 2).

For *n*-PP, the adjacent aromatic rings are connected to each other by a single C-C bond, which isolates each ring as if *n*-PP is simply the combination of several isolated benzenes. As each ring forms an aromatic sextet, *n*-PP does not have the non-Kekulé structures, and hence should display non-radical character.

By contrast, for *n*-acene and *n*-phenacene, the adjacent aromatic rings are connected to each other by sharing one common side. Due to the sawtooth arrangements of aromatic rings, it is impossible to draw the non-Kekulé structures of *n*-phenacene. Therefore, *n*-phenacene should exhibit non-radical character, showing consistency with the findings of Plasser *et al*.^26^.

On the other hand, regardless of the length of *n*-acene, the most important structure is the Kekulé structure with one aromatic sextet, the second most important structure is the non-Kekulé structure with two aromatic sextets and two unpaired *π*-electrons, and the third most important structure is the non-Kekulé structure with four aromatic sextets and four unpaired *π*-electrons. From the energy point of view (i.e., based on the previous models^52^,^53^,^54^), the non-Kekulé structures can never be dominant, and hence *n*-acene should always display non-radical character (i.e., governed by the most important Kekulé structure). However, as shown in Table 3, with the increase of *n*, the degeneracies of non-Kekulé structures (e.g., the second most important structure, the third most important structure, etc.) increase more rapidly than the degeneracy of the most important Kekulé structure. Therefore, based on our simple model, the weights of non-Kekulé structures should be increased with *n*, and may eventually be dominant for a large *n*. For a sufficiently large *n*, as the number of important non-Kekulé structures is increased, *n*-acene should display increasing polyradical character. As shown in Fig. 8, the number of fractionally occupied orbitals obtained from TAO-LDA increases with the acene length, which are indeed in support of our argument. Therefore, in addition to the energies of the structures, the degeneracies of the structures should also be taken into account in the extended Clar’s rule to qualitatively describe the polyradical character of *n*-acene^18^,^19^,^23^,^24^,^25^,^26^,^27^,^28^,^29^.

Therefore, based on our simple model, for a given number of aromatic rings *n*, the strength of radical character in the three alternant PAHs is as follows: *n*-acene > *n*-phenacene ≈ *n*-PP. To verify this, the active orbital occupation numbers (*f*_HOMO_ and *f*_LUMO_) for the lowest singlet states of these alternant PAHs calculated using TAO-LDA are shown in Table 2. Our results suggest that *n*-acene should possess radical character for *n* ≥ 6. By contrast, *n*-phenacene and *n*-PP exhibit non-radical character (even up to *n* = 20).

Based on our definition of 1D alternant PAHs, we can represent 1D alternant PAHs by a combination of the three types of arrangements in Fig. 7, and qualitatively describe their radical character. Based on the arguments above, when the adjacent aromatic rings are connected to each other by a single C-C bond (e.g., *n*-PP), the alternant PAH exhibits non-radical character, regardless of the number of these rings. By contrast, when the adjacent aromatic rings are connected to each other by sharing one common side in the linear arrangement (e.g., *n*-acene), the degeneracies of the non-Kekulé structures are quickly increased with the number of these rings, displaying an increasing polyradical character. However, when the adjacent aromatic rings are connected to each other by sharing one common side in the sawtooth arrangement (e.g., *n*-phenacene), the number of aromatic sextets in the most important Kekulé structure is increased with the number of these rings, stabilizing the alternant PAH. Therefore, for a given number of aromatic rings *n*, the more the 1D alternant PAH resembles *n*-acene, the more it displays radical character. For example, the 1D alternant PAH with zigzag edges is much less stable than that with armchair edges^12^,^29^,^30^,^58^,^59^,^60^.

Here we take the five 1D alternant PAHs (5a, 5b, 5c, 5e, and 5g) in Fig. 5 as examples. Note that the other 5-ring alternant PAHs (5d and 5f) belong to 2D alternant PAHs. To assess their radical character, it is essential to estimate the weights of the most important non-Kekulé structures relative to those of the most important Kekulé structures (see Fig. 9). First, we recognize (5a) [the linear arrangement] and (5g) [the sawtooth arrangement] as the molecules with the most and least radical character, respectively. Secondly, the strength of radical character in (5e) should be the same as that in (5g), as (5e) and (5g) both have the most important Kekulé structures with three aromatic sextets, and do not have the non-Kekulé structures. Thirdly, for (5b) and (5c), while they both have the most important non-Kekulé structures with two aromatic sextets and two unpaired *π*-electrons, the Kekulé structure of (5b) is much more stable than that of (5c) [due to an extra aromatic sextet included in the Kekulé structure of (5b)]. Therefore, the weight of the non-Kekulé structure of (5c) should be greater than that of (5b), yielding stronger radical character for (5c). Finally, based on our simple model, the strength of radical character in the five 1D alternant PAHs is as follows: (5*a*) > (5*c*) > (5*b*) > (5*e*) = (5*g*), matching reasonably well with the analysis of the TAO-LDA and RDM-CASSCF occupation numbers (see Table 1). Note that these alternant PAHs are far away from being governed by the non-Kekulé structures, and the difference of radical character among them is small. However, we can still qualitatively describe and compare the strength of radical character in these alternant PAHs by simply studying the most important Kekulé and non-Kekulé structures in our simple model.

### 2D Alternant PAHs

For 2D alternant PAHs, Pelzer *et al*. argued that the dimensionality may be an effective predictor when comparing 1D PAHs to 2D PAHs, in the sense that 1D PAHs generally exhibit more radical character than 2D PAHs^21^. However, as they commented, the above argument alone cannot explain why the longer, narrower structure (8c) exhibits less radical character than the square-shaped structure (8b) [see Table 1]. By contrast, based on our simple model, the geometry of (8c) allows more aromatic sextets to be included in the most important Kekulé structure, so the radical character of (8c) is less governed by the most important non-Kekulé structure. Therefore, (8c) exhibits less radical character than (8b). This example highlights the importance of studying the Kekulé and non-Kekulé structures in the extended Clar’s rule for qualitatively describing the radical character of alternant PAHs.

**Figure 1:**
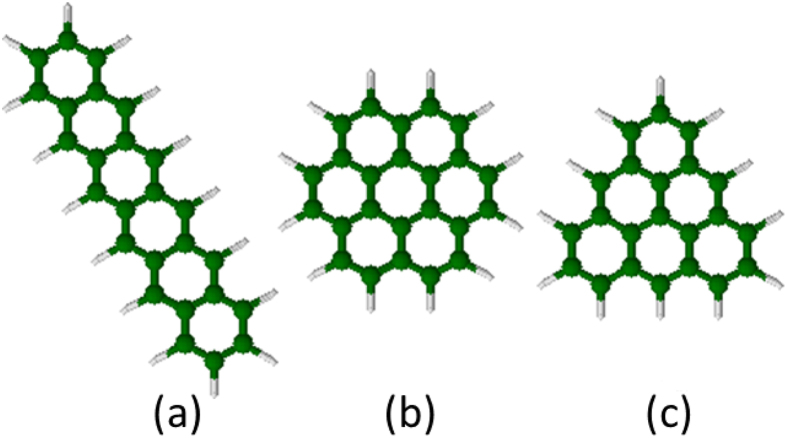
Geometries of (**a**) hexacene, (**b**) superbenzene, and (**c**) triangulene.

**Figure 2:**
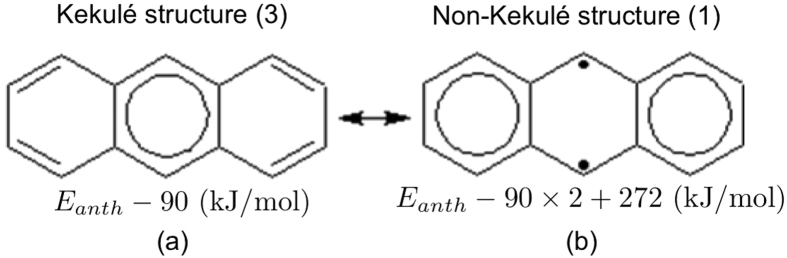
Most important Kekulé and non-Kekulé structures of anthracene. Here the aromatic sextets are marked with circles, and the unpaired *π*-electrons are marked with black dots. The energies and degeneracies (in parentheses) of the structures are shown.

**Figure 3:**
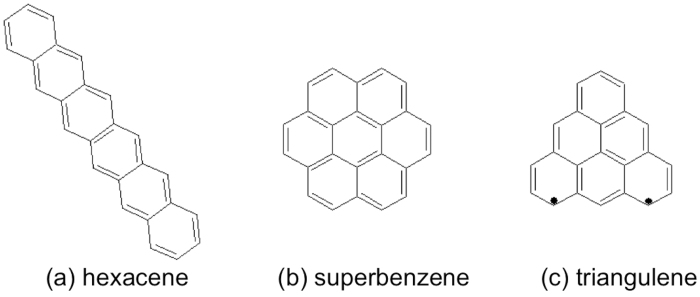
Standard structures of (**a**) hexacene, (**b**) superbenzene, and (**c**) triangulene.

**Figure 4:**
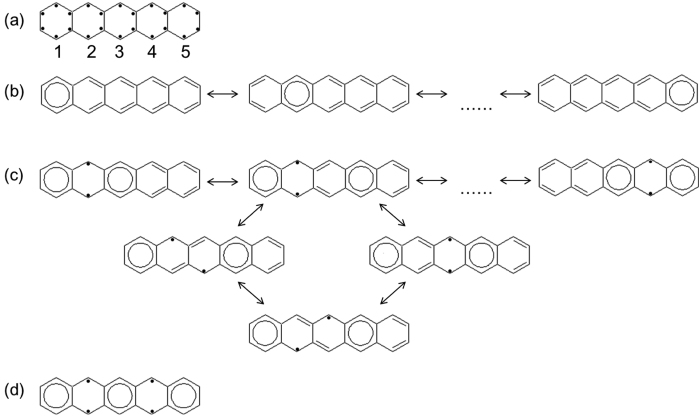
Pentacene with (**a**) all the *π*-electrons unpaired (marked with black dots), where the aromatic rings are numbered. (**b**) the most important structures (**c**) the second most important structures (**d**) the third most important structure.

**Figure 5:**
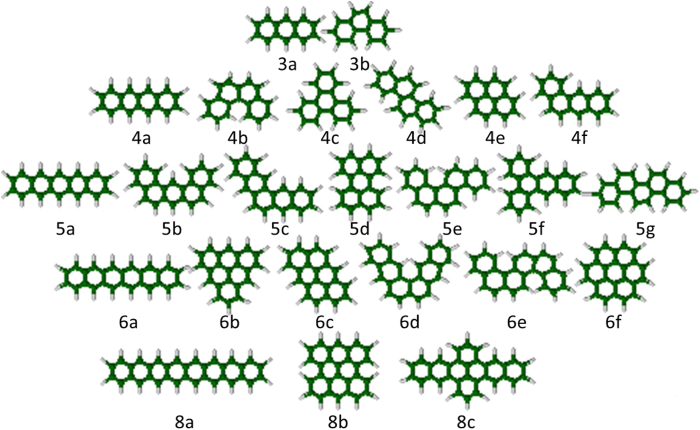
Geometries of the 24 alternant PAHs studied.

**Figure 6:**
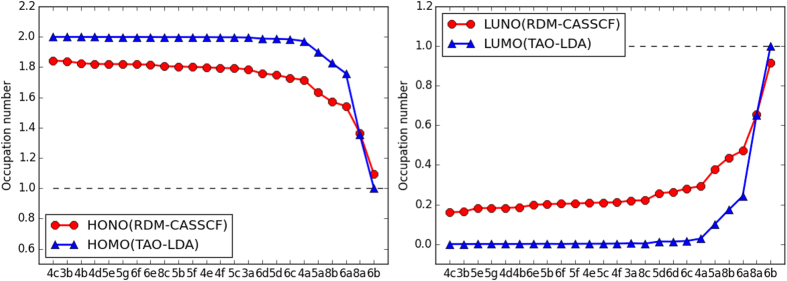
Occupation numbers of the highest occupied (*n*_HONO_) and lowest unoccupied (*n*_LUNO_) natural orbitals obtained from the RDM-CASSCF method^21^ and the occupation numbers of the highest occupied (*f*_HOMO_) and lowest unoccupied (*f*_LUMO_) molecular orbitals obtained from TAO-LDA, for the lowest singlet states of the 24 alternant PAHs studied (see Fig. 5).

**Figure 7:**
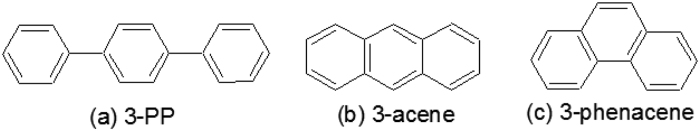
Three possible ways to arrange the aromatic rings in 1D alternant PAHs.

**Figure 8:**
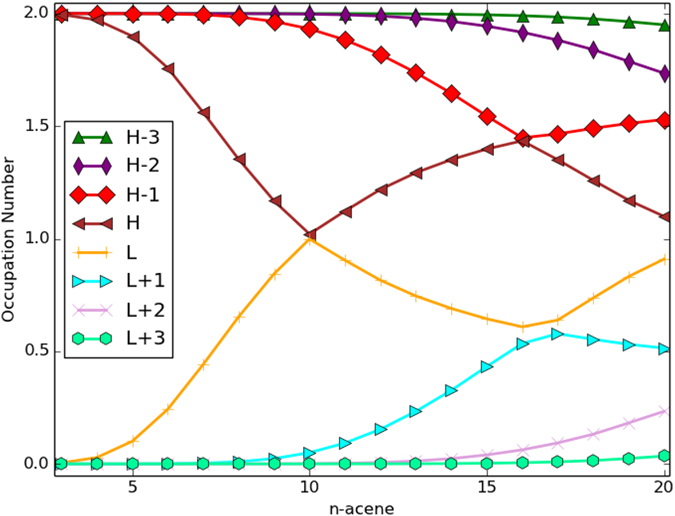
Active orbital occupation numbers ( *f*_HOMO−3_, *f*_HOMO−2_, *f*_HOMO−1_, *f*_HOMO_, *f*_LUMO_, *f*_LUMO+1_, *f*_LUMO+2_, and *f*_LUMO+3_) for the lowest singlet states of *n*-acenes as a function of the acene length *n*, calculated using TAO-LDA.

**Figure 9:**
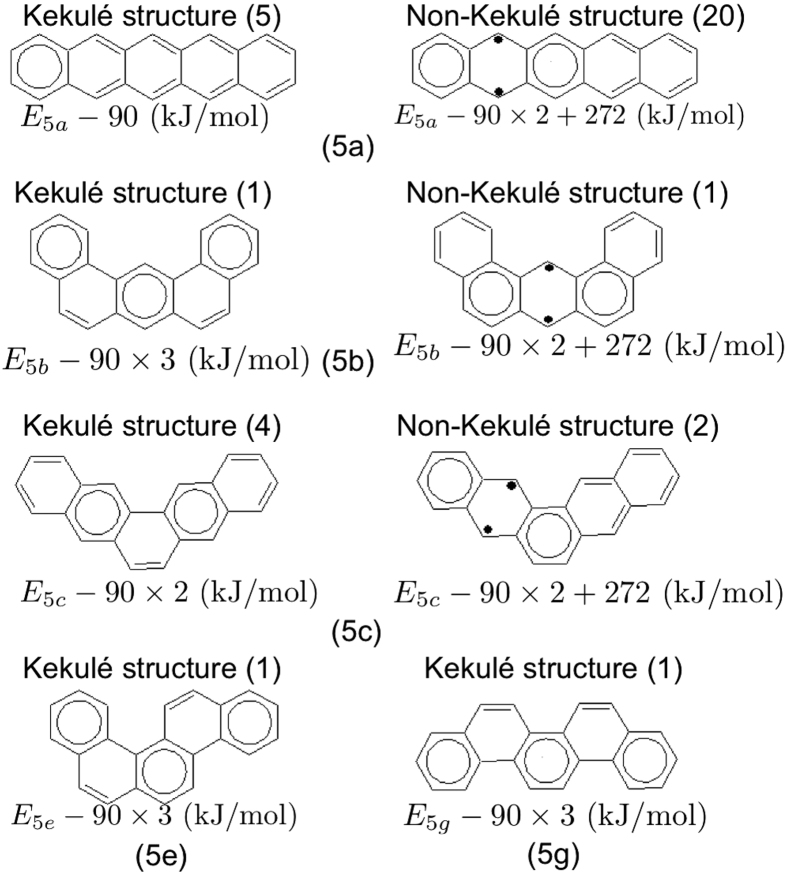
Most important Kekulé and non-Kekulé structures of 5a, 5b, 5c, 5e, and 5g. Note that 5e and 5g do not have the non-Kekulé structures. The energies and degeneracies (in parentheses) of the structures are shown.

**Figure 10:**
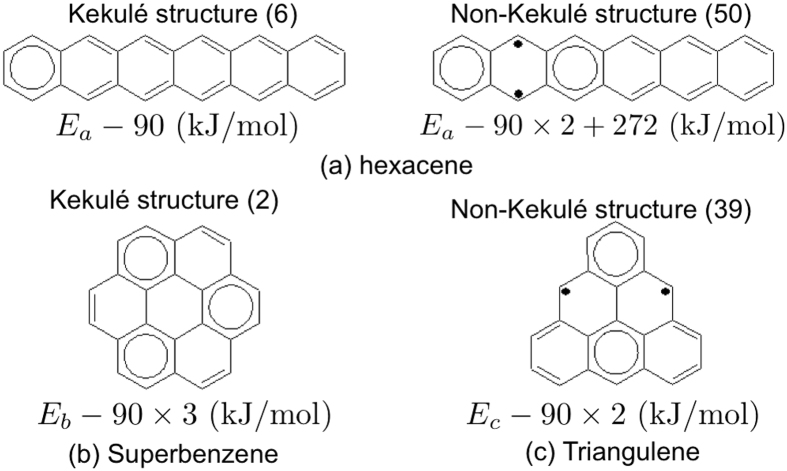
Most important Kekulé and non-Kekulé structures of hexacene, superbenzene, and triangulene. Note that superbenzene does not have the non-Kekulé structures, and triangulene does not have the Kekulé structures. The energies and degeneracies (in parentheses) of the structures are shown.

Similar arguments may be applied to the three alternant PAHs in Fig. 1. Figure 10 shows the most important Kekulé and non-Kekulé structures of hexacene, superbenzene, and triangulene. Systems with greater symmetry (e.g., superbenzene) do not necessarily exhibit stronger radical character^21^. As superbenzene does not have the non-Kekulé structures, and hence the Kekulé structures must be dominant, superbeneze exhibits non-radical character. By contrast, it is impossible to draw the Kekulé structures of triangulene, and two unpaired *π*-electrons always appear in the most important non-Kekulé structures, yielding the diradical character.

**Figure 11:**
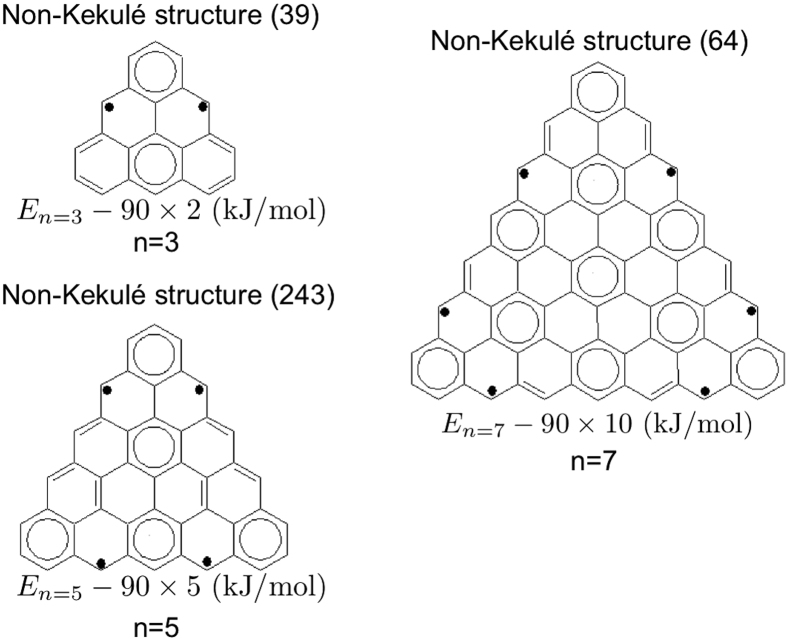
Most important non-Kekulé structures of zigzag-edged triangular graphene nanoflakes (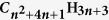) with *n* = 3, 5, and 7. Note that zigzag-edged triangular graphene nanoflakes do not have the Kekulé structures. The energies and degeneracies (in parentheses) of the structures are shown.

**Figure 12:**
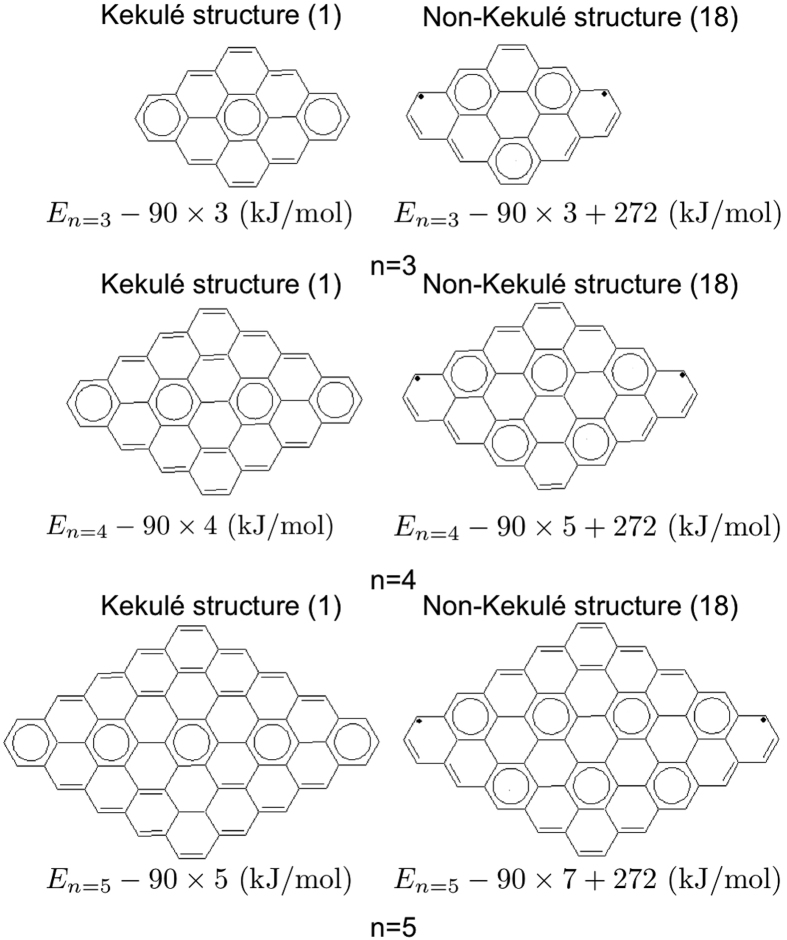
Most important Kekulé and non-Kekulé structures of zigzag-edged diamond-shaped graphene nanoflakes (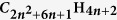) with *n* = 3, 4, and 5. The energies and degeneracies (in parentheses) of the structures are shown.

In contrast to 1D alternant PAHs, it is not practical to classify 2D alternant PAHs into groups, and formulate general rules describing their radical character, as there are too many possible geometrical arrangements of aromatic rings to study all of them. In this work, we adopt the following two types of 2D alternant PAHs: zigzag-edged triangular graphene nanoflakes (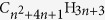) [see Fig. 11] and zigzag-edged diamond-shaped graphene nanoflakes (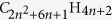) [see Fig. 12], with *n* being the number of aromatic rings at each side, as the test systems to investigate the role of Kekulé and non-Kekulé structures in the radical character of these 2D alternant PAHs.

### Zigzag-Edged Triangular Graphene Nanoflakes

Zigzag-edged triangular graphene nanoflakes (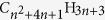) with *n* = 3, 5, 7, 9, and 11, all possess singlet ground states, based on the TAO-LDA calculations (see Table 4). Note that those with an even number of *n* are excluded, as they contain an odd number of electrons, and do not possess singlet states. Similar to triangulene (i.e., *n* = 3), zigzag-edged triangular graphene nanoflakes do not have the Kekulé structures, and hence the non-Kekulé structures must be dominant. Figure 11 shows the most important non-Kekulé structures of zigzag-edged triangular graphene nanoflakes with *n* = 3, 5, and 7. Due to the peculiar geometries of zigzag-edged triangular graphene nanoflakes, unpaired *π*-electrons always appear in the most important non-Kekulé structures (as well as the standard structures). According to Fig. 11, there are 2, 4, and 6 unpaired *π*-electrons for the *n* = 3, 5, and 7 cases, respectively. We can extend this observation, and argue that there should be (*n* − 1) unpaired *π*-electrons in the zigzag-edged triangular graphene nanoflake with *n* aromatic rings at each side. As shown in Table 4, the active orbital occupation numbers for the lowest singlet states of zigzag-edged triangular graphene nanoflakes with *n* = 3, 5, 7, 9, and 11, calculated using TAO-LDA, are indeed in support of our simple model. Therefore, zigzag-edged triangular graphene nanoflakes with longer side length should exhibit increasing polyradical character.

### Zigzag-Edged Diamond-Shaped Graphene Nanoflakes

Zigzag-edged diamond-shaped graphene nanoflakes (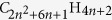) with *n* = 3–7, all have singlet ground states, based on the TAO-LDA calculations (see Table 5). Figure 12 shows the most important Kekulé and non-Kekulé structures of zigzag-edged diamond-shaped graphene nanoflakes with *n* = 3–5 and their corresponding degeneracies. For *n* = 3, as the energy of the most important Kekulé structure is lower than that of the most important non-Kekulé structure, and the degeneracy of the most important non-Kekulé structure is insufficiently large, the radical character of the molecule is mainly governed by the most important Kekulé structure, hence displaying a closed-shell singlet ground state. For the larger *n*, while the degeneracies of the most important Kekulé and non-Kekulé structures remain the same as those for *n* = 3, the energy difference between these two structures becomes smaller, and hence the weight of non-Kekulé structure becomes relatively larger. For *n* > 6, the energy of the most important non-Kekulé structure becomes even lower than that of the most important Kekulé structure, so the molecule displays strong radical character. As shown in Table 5, the active orbital occupation numbers for the lowest singlet states of zigzag-edged diamond-shaped graphene nanoflakes with *n* = 3–7, calculated using TAO-LDA, show consistency with our simple model.

## Conclusions

In conclusion, we have shown that the TAO-LDA occupation numbers are qualitatively similar to the NOONs obtained from the accurate RDM-CASSCF method, and are potentially useful for assessing the radical character of large alternant PAHs, due to its computational efficiency. Relative to the analysis of the TAO-LDA and RDM-CASSCF occupation numbers, the studies of Kekulé and non-Kekulé structures in our proposed simple model qualitatively describe the radical character of alternant PAHs, which could be useful when electronic structure calculations are infeasible due to the expensive computational cost. Our results support previous findings on the increase in radical character with increasing system size. For alternant PAHs with the same number of aromatic rings, the geometrical arrangements of aromatic rings have been shown to be influential for the radical character of alternant PAHs. For 1D alternant PAHs, the more the molecules resemble the acene series, the more they display polyradical character. For 2D alternant PAHs, it is impractical to classify the molecules into groups, and formulate general rules describing their radical character, as there are too many possible geometrical arrangements of aromatic rings to study all of them. Nevertheless, the radical character of several 2D alternant PAHs has been qualitatively described by our simple model.

## Additional Information

**How to cite this article**: Yeh, C.-N. and Chai, J.-D. Role of Kekulé and Non-Kekulé Structures in the Radical Character of Alternant Polycyclic Aromatic Hydrocarbons: A TAO-DFT Study. *Sci. Rep.* **6**, 30562; doi: 10.1038/srep30562 (2016).

## Figures and Tables

**Table 1:** Active orbital occupation numbers (*f*_HOMO_ and *f*_LUMO_) for the lowest singlet states of the 24 alternant PAHs studied (see Fig. 5), calculated using TAO-LDA.

	RDM-CASSCF		TAO-LDA		
	*n*_HONO_	*n*_LUNO_	*f*_HOMO_	*f*_LUMO_	*E*_ST_
3a	1.785	0.220	1.996	0.004	1.87
3b	1.839	0.164	2.000	0.000	2.74
4a	1.715	0.293	1.972	0.028	1.26
4f	1.794	0.211	1.997	0.003	2.04
4e	1.799	0.208	1.998	0.002	2.17
4d	1.821	0.183	1.999	0.001	2.45
4b	1.826	0.186	1.999	0.001	2.46
4c	1.843	0.160	2.000	0.000	2.82
5a	1.632	0.379	1.900	0.100	0.85
5d	1.749	0.257	1.987	0.013	1.54
5c	1.793	0.210	1.997	0.002	1.86
5f	1.801	0.205	1.998	0.002	2.15
5b	1.804	0.202	1.998	0.002	2.16
5e	1.820	0.182	1.999	0.001	2.34
5g	1.820	0.182	1.999	0.001	2.35
6b	1.095	0.916	1.000	1.000	0.28
6a	1.542	0.472	1.759	0.242	0.59
6c	1.728	0.281	1.984	0.016	1.47
6d	1.758	0.263	1.988	0.013	1.56
6e	1.816	0.198	1.999	0.002	2.23
6f	1.818	0.204	1.999	0.001	2.23
8a	1.364	0.655	1.352	0.653	0.34
8b	1.572	0.437	1.827	0.173	0.72
8c	1.806	0.221	1.998	0.003	1.87

For comparison, the corresponding natural orbital occupation numbers (*n*_HONO_ and *n*_LUNO_, respectively) obtained from the RDM-CASSCF method^21^ are shown. As the singlet-triplet energy gaps *E*_ST_ (in eV) of these alternant PAHs are positive, they all possess singlet ground states.

**Table 2:** Active orbital occupation numbers ( *f*_HOMO_ and *f*_LUMO_) for the lowest singlet states of *n*-PP, *n*-acene, and *n*-phenacene (*n* = 3–20), calculated using TAO-LDA.

*n*	*n*-PP			*n*-acene			*n*-phenacene		
	*f*_HOMO_	*f*_LUMO_	*E*_ST_	*f*_HOMO_	*f*_LUMO_	*E*_ST_	*f*_HOMO_	*f*_LUMO_	*E*_ST_
3	1.999	0.001	2.51	1.996	0.004	1.87	2.000	0.002	2.74
4	1.998	0.002	2.24	1.972	0.028	1.26	1.999	0.001	2.45
5	1.997	0.003	2.06	1.900	0.100	0.85	1.999	0.001	2.35
6	1.996	0.004	1.93	1.759	0.242	0.59	1.999	0.001	2.25
7	1.995	0.006	1.83	1.561	0.441	0.43	1.998	0.001	2.17
8	1.994	0.007	1.74	1.353	0.653	0.34	1.998	0.001	2.11
9	1.993	0.008	1.67	1.168	0.844	0.29	1.998	0.001	2.05
10	1.992	0.008	1.62	1.020	1.000	0.26	1.998	0.002	2.00
11	1.992	0.009	1.57	1.123	0.904	0.23	1.998	0.002	1.96
12	1.991	0.010	1.52	1.218	0.816	0.21	1.998	0.002	1.92
13	1.991	0.010	1.48	1.293	0.746	0.19	1.998	0.002	1.89
14	1.990	0.010	1.45	1.351	0.690	0.17	1.997	0.002	1.85
15	1.990	0.011	1.42	1.397	0.645	0.16	1.997	0.002	1.83
16	1.990	0.011	1.39	1.435	0.608	0.15	1.997	0.002	1.80
17	1.989	0.011	1.36	1.351	0.638	0.14	1.997	0.002	1.77
18	1.989	0.012	1.33	1.259	0.737	0.13	1.997	0.002	1.75
19	1.989	0.012	1.31	1.176	0.827	0.12	1.997	0.002	1.73
20	1.989	0.012	1.29	1.099	0.910	0.11	1.997	0.002	1.71

As the singlet-triplet energy gaps *E*_ST_ (in eV) of these alternant PAHs are positive, they all possess singlet ground states.

**Table 3:** Degeneracies of the top three most important structures of *n*-acene as a function of the acene length *n*.

*n*	Most important	Second most important	Third most important
3	3	1	0
4	4	6	0
5	5	20	1
6	6	50	10
7	7	105	53
8	8	196	200
9	9	336	606
10	10	540	1572
11	11	825	3630
12	12	1210	7656
13	13	1716	15015
14	14	2366	27742
15	15	3185	48763
16	16	4200	82160
17	17	5440	133484
18	18	6936	209924
19	19	8721	321091
20	20	10830	479586

Here the most important structure is the Kekulé structure with one aromatic sextet, the second most important structure is the non-Kekulé structure with two aromatic sextets and two unpaired *π*-electrons, and the third most important structure is the non-Kekulé structure with four aromatic sextets and four unpaired *π*-electrons.

**Table 4:** Active orbital occupation numbers (*f*_HOMO−5_, …, *f*_HOMO−1_, *f*_HOMO_, *f*_LUMO_, *f*_LUMO+1_, …, and *f*_LUMO+5_) for the lowest singlet states of zigzag-edged triangular graphene nanoflakes ( 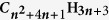 ) as a function of the side length *n*, calculated using TAO-LDA.

*n*	3	5	7	9	11
*f*_HOMO−5_	2.000	2.000	2.000	1.998	1.995
*f*_HOMO−4_	2.000	2.000	2.000	1.998	1.105
*f*_HOMO−3_	2.000	2.000	2.000	1.097	1.105
*f*_HOMO−2_	2.000	2.000	1.082	1.097	1.092
*f*_HOMO−1_	2.000	1.055	1.082	1.076	1.029
*f*_HOMO_	1.000	1.055	1.045	0.998	0.999
*f*_LUMO_	1.000	0.981	0.963	0.962	0.999
*f*_LUMO+1_	0.000	0.981	0.963	0.962	0.929
*f*_LUMO+2_	0.000	0.000	0.914	0.904	0.929
*f*_LUMO+3_	0.000	0.000	0.000	0.904	0.914
*f*_LUMO+4_	0.000	0.000	0.000	0.001	0.901
*f*_LUMO+5_	0.000	0.000	0.000	0.001	0.002
*E*_ST_	0.28	0.08	0.05	0.04	0.03

As the singlet-triplet energy gaps *E*_ST_ (in eV) of these alternant PAHs are positive, they all possess singlet ground states.

**Table 5:** Active orbital occupation numbers (*f*_HOMO−5_, …, *f*_HOMO−1_, *f*_HOMO_, *f*_LUMO_, *f*_LUMO+1_, …, and *f*_LUMO+5_) for the lowest singlet states of zigzag-edged diamond-shaped graphene nanoflakes ( 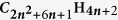 ) as a function of the side length *n*, calculated using TAO-LDA.

*n*	3	4	5	6	7
*f*_HOMO−5_	2.000	2.000	2.000	2.000	1.999
*f*_HOMO−4_	2.000	2.000	2.000	2.000	1.997
*f*_HOMO−3_	2.000	2.000	2.000	1.995	1.950
*f*_HOMO−2_	2.000	2.000	1.990	1.905	1.672
*f*_HOMO−1_	1.999	1.976	1.821	1.523	1.277
*f*_HOMO_	1.938	1.668	1.351	1.161	1.070
*f*_LUMO_	0.062	0.334	0.660	0.853	0.946
*f*_LUMO+1_	0.000	0.021	0.170	0.479	0.745
*f*_LUMO+2_	0.000	0.000	0.008	0.080	0.300
*f*_LUMO+3_	0.000	0.000	0.000	0.004	0.040
*f*_LUMO+4_	0.000	0.000	0.000	0.000	0.002
*f*_LUMO+5_	0.000	0.000	0.000	0.000	0.001
*E*_ST_	1.02	0.44	0.20	0.12	0.08

As the singlet-triplet energy gaps *E*_ST_ (in eV) of these alternant PAHs are positive, they all possess singlet ground states.
